# Atmospheric Pressure Plasma Polymerized 2-Ethyl-2-oxazoline Based Thin Films for Biomedical Purposes

**DOI:** 10.3390/polym12112679

**Published:** 2020-11-13

**Authors:** Věra Mazánková, Pavel Sťahel, Petra Matoušková, Antonín Brablec, Jan Čech, Lubomír Prokeš, Vilma Buršíková, Monika Stupavská, Marián Lehocký, Kadir Ozaltin, Petr Humpolíček, David Trunec

**Affiliations:** 1 Department of Mathematics and Physics, Faculty of Military Technology, University of Defence in Brno, Kounicova 65, 662 10 Brno, Czech Republic; 2 Institute of Physical and Applied Chemistry, Faculty of Chemistry, Brno University of Technology, Purkyňova 118, 612 00 Brno, Czech Republic; 3 Department of Physical Electronics, Faculty of Science, Masaryk University, Kotlářská 2, 611 37 Brno, Czech Republic; pstahel@physics.muni.cz (P.S.); abr92@sci.muni.cz (A.B.); cech@physics.muni.cz (J.Č.); luboprok@gmail.com (L.P.); vilmab@physics.muni.cz (V.B.); stupavska@mail.muni.cz (M.S.); trunec@physics.muni.cz (D.T.); 4 Institute of Food Science and Biotechnology, Faculty of Chemistry, Brno University of Technology, Purkyňova 118, 612 00 Brno, Czech Republic; matouskova@fch.vut.cz; 5 Centre of Polymer Systems, Tomas Bata University in Zlín, Trida Tomase Bati 5678, 760 01 Zlín, Czech Republic; lehocky@post.cz (M.L.); kadirozaltin@hotmail.com (K.O.); humpolicek@utb.cz (P.H.); 6 Faculty of Technology, Tomas Bata University in Zlín, Vavreckova 275, 760 01 Zlín, Czech Republic

**Keywords:** antibiofouling, plasma polymer, oxazoline

## Abstract

Polyoxazoline thin coatings were deposited on glass substrates using atmospheric pressure plasma polymerization from 2-ethyl-2-oxazoline vapours. The plasma polymerization was performed in dielectric barrier discharge burning in nitrogen at atmospheric pressure. The thin films stable in aqueous environments were obtained at the deposition with increased substrate temperature, which was changed from 20 ${}^{\circ }$C to 150 ${}^{\circ }$C. The thin film deposited samples were highly active against both *S. aureus* and *E. coli* strains in general. The chemical composition of polyoxazoline films was studied by FTIR and XPS, the mechanical properties of films were studied by depth sensing indentation technique and by scratch tests. The film surface properties were studied by AFM and by surface energy measurement. After tuning the deposition parameters (i.e., monomer flow rate and substrate temperature), stable films, which resist bacterial biofilm formation and have cell-repellent properties, were achieved. Such antibiofouling polyoxazoline thin films can have many potential biomedical applications.

## 1. Introduction

Poly(2-oxazoline) (POx) is a promising alternative to polyethylene glycol (PEG) for polymer functionalization of surfaces which can impart desired biochemical properties to different materials [1]. POx has attracted substantial attention recently due to its antibiofouling properties [2,3] and good biocompatibility [4]. So, polymer materials used in medicine (polypropylene, polyethylene, polytetrafluoroethylene) can be coated by POx thin layer, which can suppress the creation of bacterial biofilm on their surface. POx can be synthesized via cationic ring-opening polymerization (CROP) or by surface-initiated CROP (SI-CROP). CROP and SI-CROP are wet chemical processes using different catalysts, which have several disadvantages compared to dry processes. The most obvious is the costly removal of catalyst impurities from the resultant polymers. Other possible polymerization techniques are photocoupling [5] and grafting [6], both of which require the premodification of substrates. So, the formation of POx coatings using conventional methods is a slow and complex multistep procedure, which can be conducted only on a limited range of substrates. Recently, plasma polymerization of 2-oxazolines was used to produce robustly attached coatings on different substrates. Plasma polymerization is a one-step and substrate-independent dry process, which does not require the use of solvents or initiators and therefore it does not create liquid organic waste. So, the drawbacks of CROP are overcome by plasma polymerization of POx. Plasma polymerization is already known to be a suitable method for the deposition of many biomaterial coatings [7,8]. For the first time, plasma polymerization of POx from 2-ethyl-2-oxazoline was performed in a low pressure inductively excited pulsed radio frequency (RF) discharge [9]. Later, plasma deposition of 2-methyl-2-oxazoline and 2-ethyl-2-oxazoline was performed in low pressure RF discharge [10,11,12,13].

However, the necessity to use expensive vacuum pumping systems is the disadvantage of low pressure plasma polymerization techniques. Plasma polymerization at the atmospheric-pressure (AP) is a more cost-efficient alternative to low-pressure plasma polymerization, as it requires less sophisticated or no vacuum equipment, is energy-efficient and also offers comparatively higher deposition rates [14].

A suitable discharge type for plasma deposition at atmospheric pressure is atmospheric pressure Townsend-like discharge (APTD), which is a homogeneous dielectric barrier discharge (DBD) [15]. APTD was already used e.g., for SiO${}_{2}$ thin film deposition [16] or for deposition of organosilicon polymer films [17]. The properties of thin films deposited in APTD can be improved by increasing the substrate temperature at deposition [18]. A homogeneous DBD can be also generated in helium or neon working gases [19,20], however, nitrogen is a cheaper alternative to these noble gases.

Recently, also POx coatings were deposited using different discharge configurations at atmospheric pressure. Atmospheric pressure helium plasma jet was used for plasma polymerisation of 2-methyl-2-oxazoline on heated silicon substrates [21]. In these experiments, the POx film stability in buffer solution was substantially improved when the films were deposited at substrate temperatures above 50 ${}^{\circ }$C. Near atmospheric pressure (0.5 bar) argon DBD was used for plasma polymerization of 2-alkyl-2-oxazolines and the influence of the aliphatic side-chain length on the plasma polymerization process conditions as well as on the properties of the deposited coatings was studied [22].

In our previous study, POx thin coatings were deposited in nitrogen APTD using 2-methyl-2-oxazoline vapour as a monomer. The glass substrates were heated up to 150 ${}^{\circ }$C during the deposition process [23]. The POx films with the highest biocompatibility and the best antibiofoulding properties were obtained at the deposition with a substrate temperature of 150 ${}^{\circ }$C. From above mentioned studies follows that the deposition conditions have to be properly chosen in order to obtain the POx coatings which are stable in aqueous environments and have the best antibiofouling properties.

In the current study, POx thin films were deposited in nitrogen APTD using 2-ethyl-2-oxazoline as a monomer. The substrate temperature was changed from 20 ${}^{\circ }$C to 150 ${}^{\circ }$C at the film deposition. This temperature change led to different film properties. The film properties were compared with POx film properties deposited from 2-methyl-2-oxazoline at the same deposition conditions in our previous study [23].

## 2. Materials and Methods

### 2.1. Materials

Glass plates (soda-lime glass, 150 × 100 mm, thickness 1.1 mm) were used as substrates for deposition. 2-Ethyl-2-oxazoline (99%, Sigma-Aldrich, Munich, Germany) was used as a monomer for plasma deposition. The first set of antibacterial tests was done with *Staphylococcus aureus* (CCM 4516) and *Escherichia coli* (CCM 4517) both supplied by the Czech Collection of Microorganisms in Brno. The second set of antibacterial tests was done with *Staphylococcus epidermidis* (CCM 4418), supplied by the Czech Collection of Microorganisms in Brno. This bacterial culture was grown into commercial BHI medium (Brain Heart Infusion Broth, HiMedia, Mumbai, India). Basic Red 2 (Safranin O, Sigma-Aldrich, Munich, Germany) stain was used for biofilms visualization.

### 2.2. Plasma Deposition and Discharge Diagnostics

Plasma polymerization was performed in a custom build reactor with dielectric barrier discharge [18]. The description of the reactor, its scheme and the picture of the electrode system have been provided in our earlier works [23,24] and an only brief description will be presented here. The discharge was generated between two planar metal electrodes. The bottom electrode could be heated using a heating spiral, and the electrode temperature was measured with a thermocouple. The upper electrode was covered with glass and it was periodically moving with a speed of 0.6 cm s${}^{-1}$ above the substrate during the deposition to ensure an even greater homogeneity of the deposited film. A slit 2 mm wide in the centre of the upper electrode was used to supply working gas with the monomer to the discharge. The glass substrates were cleaned in a mixture of cyclohexane and isopropyl alcohol (1:1) and dried in airflow. Clean substrates were put into the reactor on the bottom electrode, which was then entirely covered by the substrate. The discharge gap between the substrate and the upper electrode was set to be 1.0 mm. Before starting the depositions, the discharge chamber was pumped down to a pressure of 100 Pa and then filled with nitrogen to a pressure of 101 kPa. Atmospheric pressure during the deposition was maintained by slight pumping.

Nitrogen flow with flow rates from 50 sccm to 500 sccm bubbled through the liquid 2-ethyl-2-oxazoline monomer in a glass bottle container. This flow was then mixed with the main nitrogen flow with the flow rate of 500 sccm. The temperature of the monomer was kept constant and set to 20 ${}^{\circ }$C. The monomer flow rate was determined by weighing the monomer before and after the deposition, the flow rate of the monomer was 0.033 g min${}^{-1}$ for the nitrogen flow of 60 sccm through the liquid monomer and the monomer flow rate increased linearly up 0.43 g min${}^{-1}$ at nitrogen flow of 500 sccm. High voltage with the frequency 6 kHz was used for the generation of a homogenous atmospheric pressure Townsend-like discharge (APTD) [17]. The discharge mode was checked by current-voltage measurements using an oscilloscope and by optical imaging, see bellow. The forward input power to a high voltage source was set to 55 W and it was constant in all experiments presented in this paper. The temperature of the bottom electrode was increased to a given value before the deposition. The deposition time was 23 min.

To distinguish between the natural filamentary regime of DBD and required homogeneous regime used for the deposition process, the discharge image was recorded and analysed using high-speed intensified CCD camera PI-MAX 3 (Princeton Instruments, Trenton, NJ, USA) with the shortest gating times up to 3 nanoseconds. The camera has a resolution of 1024×1024 pixels, and it was equipped with the SIGMA 105/2.8 EX DG MACRO lens (SIGMA CORPORATION, Kawasaki-shi, Japan).

### 2.3. Surface Characterization

The IR spectra of deposited films were measured by FTIR spectrometer Alpha (Bruker, Billerica, MA, USA) using a single reflection ATR module Platinum. The total surface free energy of the films was determined from measurements of contact angles between testing liquids and the film surfaces using a sessile drop technique. The acid-base theory was used for the calculation of total surface free energy. Atomic Force Microscope (AFM) Ntegra Prima (NT-MDT, Apeldoorn, The Netherlands) was used (the resolution was 512×512) to study the surface topography of the films. The measurements were performed in semi-contact mode on 10×10 μm${}^{2}$ and 5×5 μm${}^{2}$ areas of each coating with a scanning rate of 0.5 Hz. The 3D roughness parameters of the thin films were evaluated according to the ASME B46 standard using the NovaPx software (NT-MDT). The surface of the films after indentation and scratch tests was studied using the scanning probe (SPM) mode of the Hysitron TI 950 (Bruker, Minneapolis, MN, USA) instrument (the scanning rate was 1 Hz, the resolution was 1024×1024). The film thickness was measured using Dektak XT (Bruker, Tucson, AZ, USA) mechanical profilometer.

The XPS measurements were done on an ESCALAB 250Xi (Thermo Fisher Scientific, East Grinstead, UK). An X-ray beam with a power of 200 W (650 microns spot size) was used. The survey spectra were acquired with a pass energy of 50 eV and an energy step of 1 eV. High-resolution scans were acquired with a pass energy of 20 eV and an energy step of 0.1 eV. In order to compensate charges on the surface, an electron flood gun was used. Spectra were referenced to the hydrocarbon type C1s component set at a binding energy of 284.8 eV. Spectra calibration, processing and fitting routines were done using Avantage software.

### 2.4. Characterization of Mechanical Properties

The mechanical properties of the deposited films were determined using Hysitron TI 950 (Bruker, USA) nanoindentor. The load resolution of the instrument used was 1 nN and its displacement resolution was 0.04 nm. The load noise floor was less than 30 nN and the drift rate was less than 0.05 nm s${}^{-1}$. The standard Berkovich diamond indenter with tip radius of 70 nm was used for nanoindentation tests. The maximum indentation load was in the range from 0.1 to 11 mN. The mechanical parameters were determined at indentation depths <1/10 of film thickness to avoid any substrate effect. The hardness (*H*) and the effective elastic modulus ($E_{\mathrm{eff}}$) of films were calculated from loading-unloading dependences on the basis of the standard Oliver and Pharr method [25]. The effective elastic modulus could be expressed according to $E_{\mathrm{eff}}=E/\left(1-\nu^{2}\right)$, where ν and *E* are the Poisson’s ratio and Young’s modulus of the material, respectively. Several indentation modes were used to determine the mechanical properties of deposited films, namely basic quasistatic indentation tests (trapeozid load function based on 5 s loading, 2 s creep and 5 s unloading), quasistatic nanoindentation tests with 20 partial unloading segments and nanodynamic indentation (nanoDMA) in constant strain rate measuring mode. The nanoDMA indentation tests were carried out by superimposing a sinusoidal load with a small amplitude (30 μN to 0.2 mN) and frequency of 220 Hz on the quasistatic loading curve. Each indentation test was repeated at least 10 times. The TI 950 instrument was also equipped with transducer, which enabled to carry out scratch tests at nanoscale up to normal loads of 11 mN. The radius of conical diamond indenter used in case of scratch tests was approx. 900 nm and its opening angle was 45${}^{\circ }$. The maximum scratch load was in the region from 0.30 to 11 mN. Two types of tests were applied: tests with linearly increasing load (7 μN s${}^{-1}$) in order to find the critical load $L_{c}$ for creation of scratch induced cracks or delaminations and tests with constant load in order to determine the friction coefficient CoF. Each scratch test was repeated 4 times. A tilt correction algorithm was used to eliminate the influence of the tilt of the tested samples.

### 2.5. Antibacterial Tests

The first set of antibacterial tests was performed according to modified ISO 22196 standard. UV-radiation was utilized for sample sterilization with 30 min duration of the irradiation. The used wavelength was 258 nm. For antibacterial performance study, gram-positive *Staphylococcus aureus* (CCM 4516) and gram-negative *Escherichia coli* (CCM 4517) bacterial strains were used in our experiments. Used bacterial suspensions (*E. coli* $2.4\times 10^{6}$ CFU mL${}^{-1}$; *S. aureus* $1.7\times 10^{5}$ CFU mL${}^{-1}$) were made in 1/500 Nutrient broth (HiMedia laboratories, Mumbai, India). The bacterial suspension was dispensed on the sample surface with dimensions of 10 mm by 10 mm in the volume of 100 μL and the sample was consequently covered with the foil (dimensions 25 mm × 25 mm) made by polypropylene. Samples with foils were cultivated at the temperature of 35 ${}^{\circ }$C and 95% relative humidity for 24 h. After the incubation time, polypropylene foil was taken out of the samples and each separate sample was completely washed by SCDLP (Soybean, Casein, Digest, Lecithin, Polysorbate) broth purchased from (HiMedia laboratories, India), which was subsequently collected. Finally, the pour plate culture method (PCA, HiMedia laboratories, India) was used for the viable bacteria count determination.

The second set of antibacterial tests was performed using *Staphylococcus epidermidis*. The bacterial culture was grown into BHI medium and the temperature for microorganism cultivation was 37 ${}^{\circ }$C. After 24 h incubation, *S. epidermidis* was diluted with a new sterile medium to $1\times 10^{8}$ CFU per mL based on turbidity (NanoPhotometer™ P300, Implen, Munich, Germany). Then *S. epidermidis* suspension (500 μL) was added on each sample. The bacteria on the sample surfaces were incubated for 24 h to allow the formation of biofilms. After incubation, all samples were washed twice with Milli-Q water to remove any loosely bound biofilm. For biofilms visualization, 200 μL of Basic Red 2 stain was used. The excess of Basic Red 2 stain was then washed off and the results were evaluated using optical microscopy (Optical microscope Intraco Micro LM 666 PC/*∞* with Dino-Capture 2.0 software, Tachlovice, Czech Republic). Samples were imaged at least fifteen times each at random points and the surface area covered by bacteria was quantified using images.

### 2.6. Cytocompatibility Test

The mouse embryonic fibroblast continuous cell line (NIH/3T3, ATCC^®^ CRL-1658™, Teddington, UK) was used for cytocompatibility test according to the EN ISO 10993-5 standard, with modification. As a culture medium, the ATCC-formulated Dulbecco’s Modified Eagle’s Medium (BioSera, Nuaille, France), containing 10% calf serum (BioSera, France) and Penicillin/Streptomycin at 100 U mL${}^{-1}$ (PAA Laboratories GmbH, Pasching, Austria) was used. The tested samples were prepared with a dimension of 10 mm × 10 mm and sterilized by UV–radiation (wavelength of 258 nm) for 30 min and placed into the 24 well-plate. The cells were seeded onto the samples in the concentration of $1\times 10^{4}$ for an hour for adhesion of the cells. After the pre-cultivation, a sufficient amount of the medium was added and incubated for 48 h at 37 ${}^{\circ }$C. The changes in cell morphology were observed with an inverted fluorescent microscope (Olympus, IX 81). To assess the cytotoxic effect, an MTT assay (Duchefa, Biochemie, Haarlem, The Netherlands) was performed. The absorbance was measured by an Infinite M200 Pro NanoQuant absorbance reader (Tecan, Männedorf, Switzerland). All tests were performed in triplicates.

## 3. Results

The POx films were deposited in two series. In the first series, the films were deposited at substrate temperatures of 20, 60, 90, 120 and 150 ${}^{\circ }$C and at nitrogen flow of 100 sccm through the monomer. In the second series, the films were deposited at nitrogen flow through the monomer of 60, 100, 140, 200 and 500 sccm and at the substrate temperature of 120 ${}^{\circ }$C. All deposited films were tested for the film stability in aqueous environments, antibacterial properties and cytocompatibility. The most suitable film for biomedical purposes was selected according to the results of these tests. The selected film was then characterized by FTIR spectroscopy, XPS, AFM, depth sensing indentation technique and surface energy measurement.

### 3.1. Discharge Diagnostics

**Figure 1:**
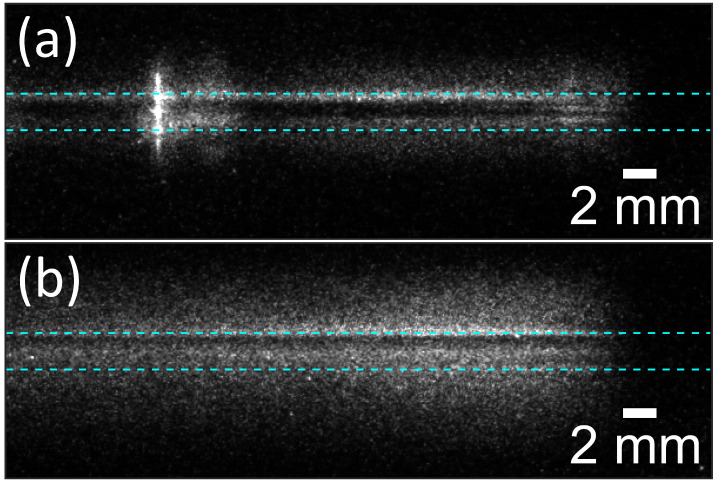
The single-shot images of the discharge half-period of the discharge generated in the nitrogen-monomer mixture: (**a**) transient homogeneous-filamentary regime; (**b**) homogeneous regime without filaments. The dashed blue lines indicate the position of the surface of the dielectric plate covering the upper electrode, and the position of the bottom electrode, which is covered by the glass substrate.

The high-quality film deposition requires the operation of the discharge in a homogeneous regime of operation. The homogeneous, i.e., the spatially unconstricted, regime of operation manifests itself through a typical current hump; however, the reliable confirmation of the discharge regime is only possible using a high-speed single-shot imaging of the discharge. The discharge imaging was performed using a fast-gated single-shot acquisition of the discharge emission in the discharge gap. Phase-locking of the camera digital delay generator to the applied high voltage enabled the acquisition of a single half-period of the discharge and subsequent analysis for the presence of the bright filament channels. The comparison of regimes is given in Figure 1 for a homogeneous and transient homogeneous-filamentary mode of operation of the discharge in the nitrogen-monomer mixture. The line-of-sight represents the side view through the discharge gap along the electrodes.

The emission of the discharge in the homogeneous regime manifests itself as the bright and dark layers of discharge emission along the surfaces of the electrodes, see Figure 1b. In between the electrodes, no filaments are visible. Contrary, in the transient regime, the bright and constricted discharge channel, i.e., filament, could be identified in the discharge gap, see Figure 1a. Images of both regimes are influenced by the “halo” at the position of the electrodes. This artefact, however, does not prevent reliable distinguish of the discharge regime. The “halo” is the artefact of the non-telecentric optical imaging system used for close-up (macro) photography at a shallow depth of field (parallax error). Also, the glass dielectric plates covering electrodes serve as optical waveguides and cause a discharge emission mirroring on their surfaces.

### 3.2. Film Thickness and Film Stability in Water

The film thicknesses are given in Table 1. POx film behavior in aqueous solvents is important for biologically related applications such as biofouling. The stability of the films in aqueous environments was tested by measuring changes in film thickness after film immersion in water for 48 h. It was found that the POx films deposited at substrate temperatures up to 90 ${}^{\circ }$C can be quickly washed by water from the substrate. Table 1 shows the percentage of film thickness loss for films prepared using substrate temperature of 20, 60, 90, 120 and 150 ${}^{\circ }$C.

These results are in agreement with our previous results [23], where the POx films plasma polymerized from 2-methyl-2-oxazoline at lower substrate temperatures (up to 90 ${}^{\circ }$C) were also soluble in water. This lack of stability in aqueous solutions was also observed at the POx film deposition using helium plasma jet [21] when POx films were deposited at substrate temperatures up to 50 ${}^{\circ }$C. The POx film stability was also studied at RF plasma polymerization [12], where the thickness loss after 24 h immersion in water decreases from 98% to 39% with increasing RF power from 10 W to 40 W. So, the POx film stability is enhanced substantially using elevated substrate temperature or increased RF power due to the increase in crosslinking in the film caused by heating or ion bombardment.

### 3.3. Antibacterial Properties

The first set of antibacterial tests was done using *S. aureus* and *E. coli* according to the ISO 22196 procedure. Antibacterial activity of the coated glass samples against *S. aureus* and *E. coli* strains are listed in Table 2. The reference substrate glass was open to both gram positive and negative bacterial contamination and did not perform any antibacterial effect, as expected, and counted viable gram positive *S. aureus* and gram negative *E. coli* level were found almost the same.

The thin film deposited samples were highly active against both *S. aureus* and *E. coli* strains in general, which refers to both the antibacterial effect of the coating material and a successful coating process under each flow rate and temperature. The samples coated under 60, 100 and 200 sccm flow rate and at 20, 60 and 90 ${}^{\circ }$C substrate temperature were shown completely antibacterial effect with zero bacterial strain per cm${}^{2}$. A few bacterial strains of 1.7 CFU/cm${}^{2}$ of *S. aureus* and $2.4\times 10^{3}$ CFU/cm${}^{2}$ of *E. coli* were counted for the sample coated under 140 sccm flow rate. Similarly, the sample coated under 500 sccm was performed the same activity with 1.7 CFU/cm${}^{2}$ of *S. aureus* and 3.3 CFU/cm${}^{2}$ of *E. coli*. By means of processing temperature, samples coated at 120 and 150 ${}^{\circ }$C were displayed $5.1\times 10^{3}$ CFU/cm${}^{2}$ and $1.1\times 10^{3}$ CFU/cm${}^{2}$ of *E. coli* strains but they were both fully effective against *S. aureus*. Due to the coating material being the same for each glass substrates, only the coating performance, hence, homogeneity and stability of the material on the substrates was the manner of the antibacterial activity. A small difference of counted bacterial strains also depends on the surface characteristics, such as film homogeneity, wettability, roughness, charge density and functionality, cell-wall compositions of the bacterial strains and physicochemical characteristics with an efficiency with the releasing performance of the coated material from the substrate. Nevertheless, 6 of the 10 coated samples were performed completely antibacterial activity against both gram positive and negative strains, which means the flow rate of 60, 100 and 200 sccm and the temperature of 20, 60 and 90 ${}^{\circ }$C were the optimum settings for the coating process.

**Figure 2:**
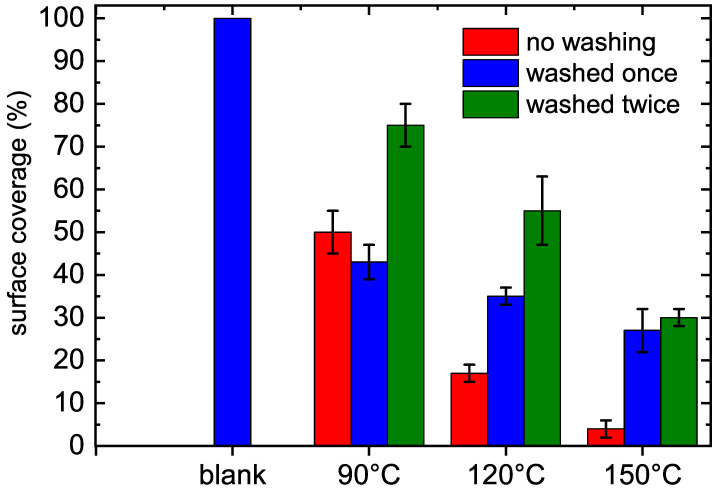
Bacteria *S. epidermidis* surface coverage area (percent) formed on POx films deposted at different substrate temperatures.

The second set of antibacterial tests was done using *S. epidermidis*, which was also used in previous studies [10,12]. Only stable POx films deposited at substrate temperatures above 60 ${}^{\circ }$C were used for these tests. The experiments were repeated twice using two identical series. Before adding the microorganism and incubation, one third of the samples was washed once, the second third was washed twice with sterile Milli-Q water in order to test the stability of prepared POx films. The last third of samples was not washed. The results of bacteria surface coverage are shown in Figure 2. It was observed in the microscopy images that *S. epidermidis* were not able to form a biofilm on POx films, only individual or small colonies were observed. In comparison, the bacteria growing on the untreated surface formed a biofilm. The possible explanations of antibiofouling properties can be found in recent review [26].

The POx films deposited from 2-methyl-2-oxazoline at the same deposition conditions exhibited the same antibacterial activity against both *S. aureus* and *E. coli* strains as presented films deposited at the flow rate of 60, 100 and 200 sccm and the temperature of 20, 60 and 90 ${}^{\circ }$C, which are even slightly better against *E. coli* strain. On the other hand, presented POx films are less effective against *S. epidermidis*, the bacterial surface coverage on POx films deposited from 2-ethyl-2-oxazoline at 90 ${}^{\circ }$C is 50%, whereas it is only 8% on films deposited from 2-methyl-2-oxazoline.

### 3.4. Cytocompatibility Results

**Figure 3:**
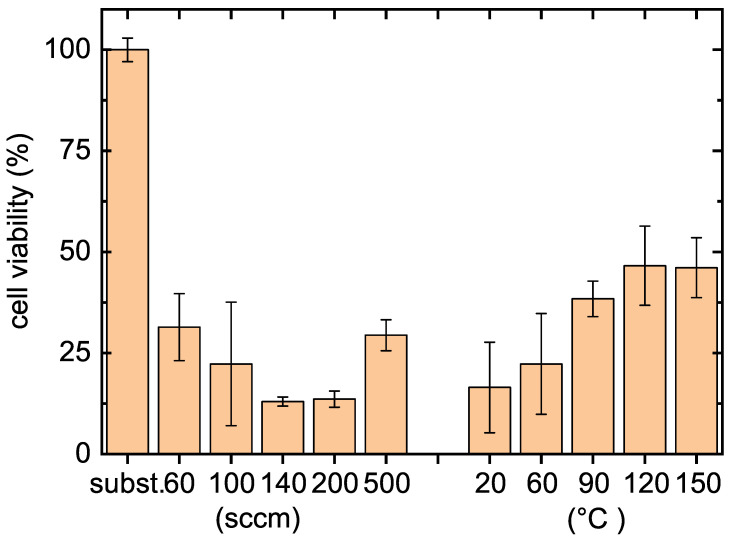
In vitro cytocompatibility results of tested POx films deposited at different substrate temperatures and at different flow rates through the monomer. Left side of graph—films deposited at different flow rates through the monomer; right side of graph—films deposited at different substrate temperatures.

In vitro cytocompatibility results were obtained using mouse embryonic fibroblast (NIH/3T3) cells. The duration of the cell interaction with tested samples was 48 h. Obtained results are presented in Figure 3, where it can be seen that cell viability for the film coated samples is significantly lower than in case of the bare glass sample which was used as a reference in our study.

This refers to the cytotoxicity of the coated material, however, the cytotoxic effects of the samples are still different. These results represent the main difference between the POx films deposited from 2-ethyl-2-oxazoline and from 2-methyl-2-oxazoline at the same deposition conditions. The POx films deposited from 2-methyl-2-oxazoline promote cell viability compared to the blank glass substrate, whereas the cells do not adhere on the POx films deposited from 2-ethyl-2-oxazoline. Such cell-repellent behavior was also observed on POx films deposited from 2-ethyl-2-oxazoline in low pressure RF discharge [9].

The above presented results of film stability in aqueous solvents, antibacterial properties and cytocompatibility show that the film deposited at the substrate temperature of 90 ${}^{\circ }$C and at flow rate of 100 sccm is the most suitable POx film for biomedical applications. The further analyses were performed for the POx film deposited just at these conditions.

### 3.5. Surface Characterization

The contact angles between the test liquids and POx film were measured in order to determine the total surface free energy using the sessile drop technique. Three test liquids were used—distilled water, glycerol and diiodomethane (CH${}_{2}$I${}_{2}$). The acid-base theory with multiple regression [27] was used to calculate the total surface free energy and its components—the Lifshitz–van der Walls (LW) interaction component and the acid–base (AB) interaction component. The surface free energy and its above mentioned components of POx film are given in Table 3.

**Figure 4:**
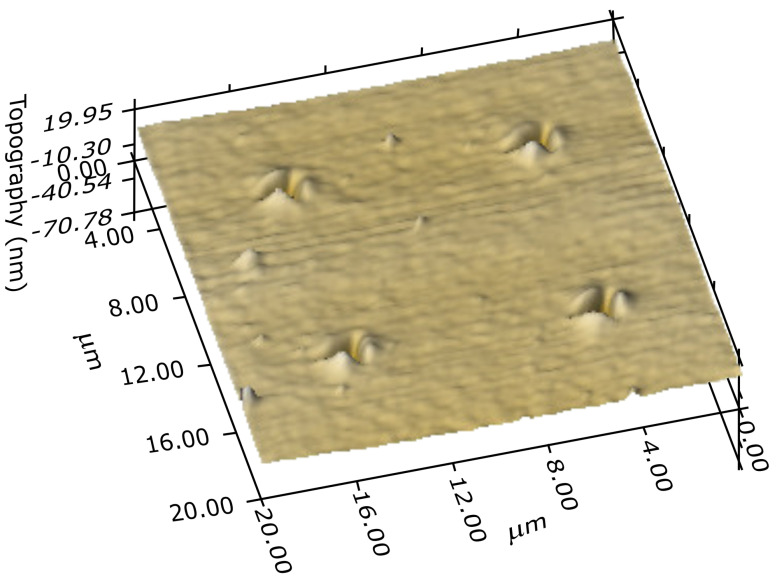
3D image of the film surface with four indentation prints made at 3 mN.

The POx film deposited at substrate temperature of 90 ${}^{\circ }$C was hydrophilic, its surface energy is 44.2 mJ m${}^{-2}$. This surface free energy is slightly lower than the surface free energy of POx films polymerized from 2-methyl-2-oxazoline in our previous experiments, which was 50.3 mJ m${}^{-2}$ [23]. The hardness of POx film was (0.60 ± 0.10) GPa and the effective elastic modulus was (11 ± 2) GPa, which are the same values as for POx films deposited from 2-methyl-2-oxazoline [23]. From the point of view of mechanical properties, the film exhibited polymer-like viscoelastic character. The hardness and elastic modulus values of the film were significantly higher than that of the common polymer materials. Moreover, the film showed excellent scratch and delamination resistance. The POx film did not exhibit indentation or scratch induced cracking or delamination up to the maximum (11 mN) of the indentation or normal scratch loads used. 3D image of the film surface with indentation prints made at 3 mN maximum indentation load is shown in Figure 4.

**Figure 5:**
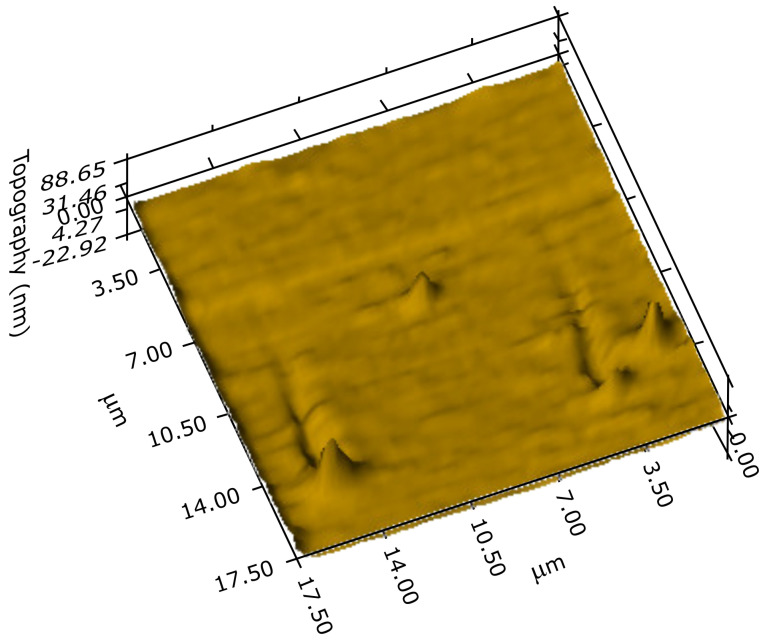
3D image of two scratch lines made with ramping normal load linearly increasing up to 0.3 mN.

No cracks or delamination effects can be seen, only indentation induced pile-ups around the indentation prints characteristic for relatively soft viscoelastic materials may be observed. The same behaviour was observed during the scratch tests, see examples of the scratch lines made with ramping normal load linearly increasing up to 0.3 mN in Figure 5, only viscoplastic deformation occurred with characteristic pile-ups, however, without cracking or delamination.

**Figure 6:**
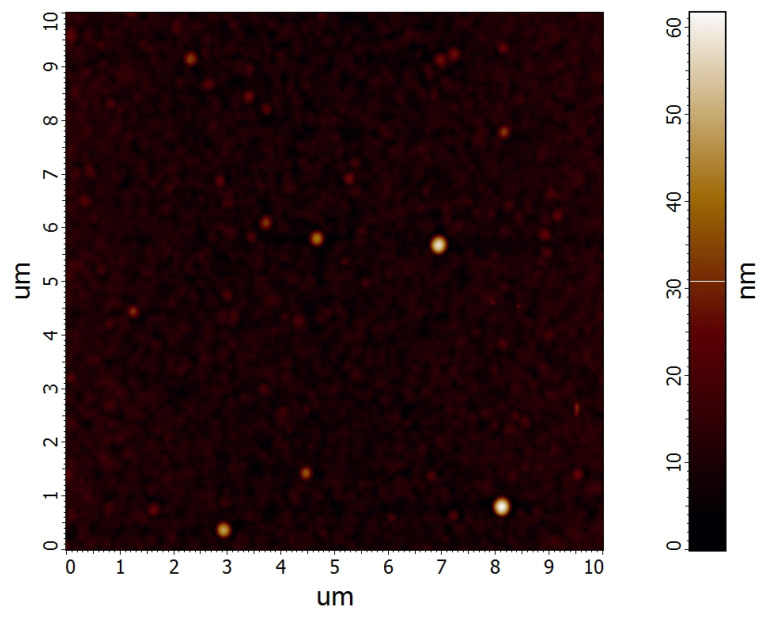
AFM image of the surface of the studied POx film.

The viscoelastic behaviour of the film may be characterised using the storage modulus $E^{\prime }$, loss modulus $E^{\prime \prime }$ and loss factor tanϕ. The storage modulus $E^{\prime }$, which is the measure of the energy stored and recovered during the loading period, was (26 ± 2) GPa and the loss modulus $E^{\prime \prime }$, which is the measure of the energy dissipated in the studied material during the loading period, was (1.4 ± 0.2) GPa. The loss factor tanϕ, which is the measure of the viscoelastic behavior of materials [28] was 0.045 ± 0.005. The studied film exhibited relatively low tanϕ value indicating a predominantly elastic behaviour (low damping) of the film material. The surface topography of the studied film is shown in Figure 6.

The root mean square (RMS) roughness of the film was (3.2 ± 0.1) nm and the average roughness was (2.1 ± 0.1) nm. The friction coefficient obtained using the constant load scratch test was 0.3.

**Figure 7:**
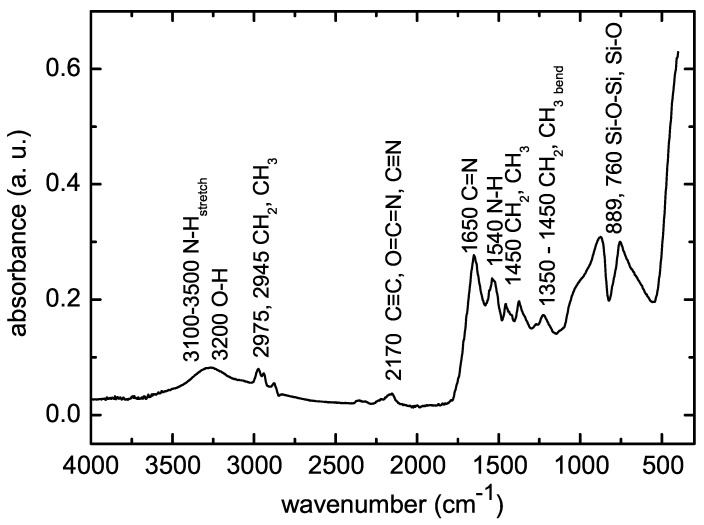
FTIR spectrum of POx thin film deposited at the substrate temperature of 90 ${}^{\circ }$C.

The FTIR spectrum of deposited POx thin film is shown in Figure 7.

Broad absorption band in the range 3000–3600 cm${}^{-1}$ consists of several peaks belonging to OH, NH and NH${}_{2}$ groups. The bands at 2975 cm${}^{-1}$, 2945 cm${}^{-1}$, 1450 cm${}^{-1}$ and 1370 cm${}^{-1}$ are characteristic for vibrations of CH${}_{3}$ and CH${}_{2}$ groups. The band at 2170 cm${}^{-1}$ can be attributed to alkyne C≡C and/or isocyanate O=C=N and nitrile C≡N. Such chemical bonds are not present at traditional polymerization of oxazolines and they can be attributed to fragmentation and recombination of the oxazoline monomer during plasma polymerization. The band between 1790 cm${}^{-1}$ and 1590 cm${}^{-1}$ is characteristic for stretching vibration C=N bond constituting the oxazoline ring. Its presence in the IR spectrum indicates the presence of oxazoline rings in deposited films. The band around 1550 cm${}^{-1}$ belongs to N-H bonds. Finally, the bands below 1000 cm${}^{-1}$ belong to Si-O-Si or Si-O bonds from substrate glass. The FTIR spectra measured on POx films deposited at different substrate temperatures and different monomer flow rates did not show significant differences.

The atomic composition of prepared POx film determined by XPS is shown in Table 4.

**Figure 8:**
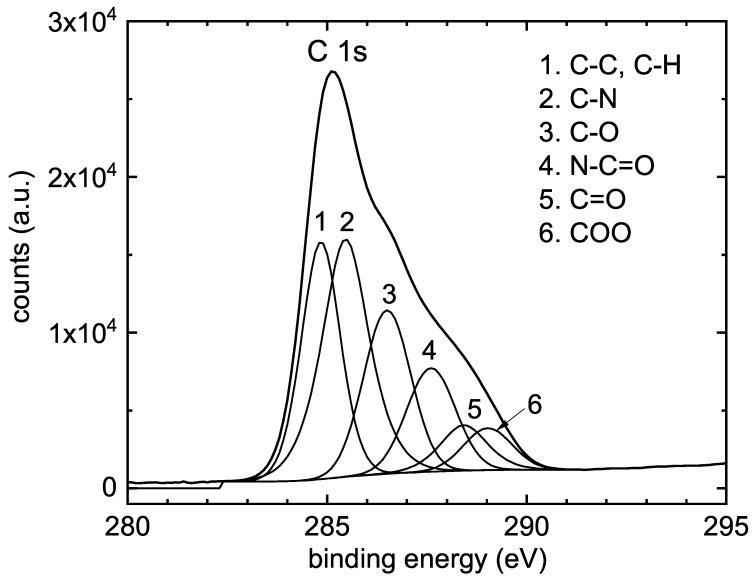
Curve fitting analysis of the high-resolution XPS C1s spectrum of POx film deposited at the substrate temperature of 90 ${}^{\circ }$C.

The nitrogen content in the POx film increased and the carbon content decreased compared to nitrogen and carbon content in 2-ethyl-2-oxazoline. However, the nitrogen content is almost two times lower than nitrogen content in POx films deposited from 2-methyl-2-oxazoline at the same deposition conditions [23]. This fact could explain lower cytocompatibility of POx films deposited from 2-ethyl-2-oxazoline, because better cytocompatibility was observed at nitrogen rich surfaces [29]. A detailed analysis of the high-resolution C1s peaks provided additional information on the chemistry changes on the surface during the film deposition process. The C1s peak of POx film was deconvoluted into six components corresponding to C-C/C-H (284.7 eV), C-N (285.4 eV), C-O (286.3 eV), N-C=O (287.7 eV), C=O (288.2 eV) and COO (289.1 eV) bonds, see Figure 8.

Thus, XPS results in addition to FTIR spectroscopy confirmed the formation of compact surface polymer layer. FTIR also revealed a partial fragmentation and recombination of the oxazoline monomer during plasma polymerization. This was not proved by XPS. A possible explanation might lay in the different information depth of both methods. FTIR is a “bulk” analytical technique collecting chemical information from micrometer range. On the other hand, the information depth endowed by the XPS, is limited only to a few nanometers.

## 4. Discussion and Conclusions

The poly(2-oxazoline) thin films were deposited on glass substrates in atmospheric pressure Townsend-like dielectric barrier discharge. The discharge burned in nitrogen, which was used as the working gas. 2-ethyl-2-oxazoline vapours were admixed to the nitrogen flow and used as the monomer. High-speed single-shot imaging of the discharge showed that a homogeneous discharge was burning at this gas composition. The increase of substrate temperature during the film deposition led to the improvement of film properties, especially to the improvement of the film stability in water. The films deposited at substrate temperatures of 20 ${}^{\circ }$C and 60 ${}^{\circ }$C were soluble in water, the films deposited at substrate temperatures of 90 ${}^{\circ }$C and higher lost less than 50% of their thickness after 48 h immersion in water. This thickness loss is comparable with results from other study of POx film plasma polymerisation [12]. The deposited films were smooth and without any pinholes. The FTIR spectra showed that some oxazoline rings are still present in the plasma polymer in contrast to classical oxazoline polymerization. The retention of the oxazoline ring is assumed to be highly beneficial for selected biomedical applications. On the other hand, some fragmentation and recombination of the oxazoline monomer was also observed in FTIR spectra. The same results from FTIR were also observed at 2-oxazoline plasma polymerization in low pressure RF discharge [10]. The POx films deposited from 2-ethyl-2-oxazoline in this study contain two times fewer nitrogen atoms than the POx films deposited from 2-methyl-2-oxazoline at the same deposition conditions in our previous study [23]. Also the carbon concentration is higher and oxygen concentration is lower in present films compared to the films deposited from 2-methyl-2-oxazoline. The N:C elemental ratio (0.31) observed in films deposited in nitrogen APTD in this study is slightly higher than the N:C ratio (0.20–0.10) observed in POx films deposited in RF discharge [12]. The deposited films were cell-repellent, again in agreement with findings in the study of other authors [9]. The POx films deposited from 2-methyl-2-oxazoline in our previous study promote the cell viability compared to present films, this is the main difference in cytocompatibility properties of both POx films. This could be explained by lower nitrogen content in present films. All deposited films exhibited excellent antibacterial properties against all bacterial strains used for antibacterial tests. Deposited films could be used as coatings with antibacterial and antibiofouling properties for biomedical applications.

## Figures and Tables

**Table 1:** The thickness and stability of POx films deposited at different substrate temperatures and at different flow rates through the monomer. The samples marked with temperature were deposited at this substrate temperature and the flow rate of 100 sccm. The samples marked with flow rate were deposited at this flow rate and the substrate temperature of 120 ${}^{\circ }$C.

Sample	Thickness (μm)	Thickness (μm)	Loss (%)
	as Deposited	after 48 h in Water	
20 ${}^{\circ }$C	0.61	0	100
60 ${}^{\circ }$C	0.60	0	100
90 ${}^{\circ }$C	1.33	0.84	37
120 ${}^{\circ }$C	2.14	1.26	41
150 ${}^{\circ }$C	0.65	0.46	29
60 sccm	1.15	0.75	35
100 sccm	2.08	1.48	29
140 sccm	0.68	0.32	53
200 sccm	0.96	0.60	38
500 sccm	1.21	1.06	22

**Table 2:** Antibacterial activity results of studied POx films. Substrate marks bare glass substrate. The samples marked with temperature were deposited at this substrate temperature and the flow rate of 100 sccm. The samples marked with flow rate were deposited at this flow rate and the substrate temperature of 120 ${}^{\circ }$C.

Sample	*S. aureus* CCM 2022 (CFU/cm${}^{2}$)	*E. coli* CCM 4517 (CFU/cm${}^{2}$)
substrate	$3.6\times 10^{4}$	$4.4\times 10^{4}$
20 ${}^{\circ }$C	<1	<1
60 ${}^{\circ }$C	<1	<1
90 ${}^{\circ }$C	<1	<1
120 ${}^{\circ }$C	<1	$5.1\times 10^{3}$
150 ${}^{\circ }$C	<1	$1.1\times 10^{3}$
60 sccm	<1	<1
100 sccm	<1	<1
140 sccm	1.7	$2.4\times 10^{3}$
200 sccm	<1	<1
500 sccm	1.7	3.3

**Table 3:** The contact angles for different liquids and surface free energy and its components of bare substrate and POx films deposited at at substrate temperature of 90 ${}^{\circ }$C and at flow rate of 100 sccm.

Sample	Contact Angle (${}^{\circ }$)			Surface Free Energy (mJ/m${}^{2}$)		
	CH${}_{2}$I${}_{2}$	glycerol	water	total	LW	AB
substrate	59.8 ± 1.2	35.5 ± 2.0	33.4 ± 2.3	52.6 ± 1.0	28.7 ± 0.7	23.9 ± 2.0
90 ${}^{\circ }$C	37.8 ± 2.0	14.7 ± 2.9	24.1 ± 3.8	44.2 ± 7.0	40.7 ± 0.9	3.5 ± 3.0

**Table 4:** Atomic composition (at%) and concentration (%) of chemical bonds for POx film deposited at substrate temperature of 90 ${}^{\circ }$C and at flow rate of 100 sccm.

Sample	N	C	O	C-C	C-N	C=O	C-O	COO	N-C=O
90 ${}^{\circ }$C	21	66	13	24	30	6	21	4	14
